# Imaging Characteristics, Tissue Distribution, and Spread of a Novel Oncolytic Vaccinia Virus Carrying the Human Sodium Iodide Symporter

**DOI:** 10.1371/journal.pone.0041647

**Published:** 2012-08-17

**Authors:** Dana Haddad, Chun-Hao Chen, Sean Carlin, Gerd Silberhumer, Nanhai G. Chen, Qian Zhang, Valerie Longo, Susanne G. Carpenter, Arjun Mittra, Joshua Carson, Joyce Au, Mithat Gonen, Pat B. Zanzonico, Aladar A. Szalay, Yuman Fong

**Affiliations:** 1 Department of Surgery, Memorial Sloan-Kettering Cancer Center, New York, New York, United States of America; 2 Department of Biochemistry, University of Wuerzburg, Wuerzburg, Bavaria, Germany; 3 Radiopharmaceutical Chemistry Service, Department of Radiology, Memorial Sloan-Kettering Cancer Center, New York, New York, United States of America; 4 Genelux Corporation, San Diego Science Center, San Diego, California, United States of America; 5 Departments of Medical Physics and Radiology, Memorial Sloan-Kettering Cancer Center, New York, New York, United States of America; 6 Department of Epidemiology and Biostatistics, Memorial Sloan-Kettering Cancer Center, New York, New York, United States of America; 7 Department of Radiation Oncology, University of California, San Diego, California, United States of America; University of Texas, M.D. Anderson Cancer Center, United States of America

## Abstract

**Introduction:**

Oncolytic viruses show promise for treating cancer. However, to assess therapy and potential toxicity, a noninvasive imaging modality is needed. This study aims to determine the *in vivo* biodistribution, and imaging and timing characteristics of a vaccinia virus, GLV-1h153, encoding the human sodium iodide symporter (hNIS.

**Methods:**

GLV-1h153 was modified from GLV-1h68 to encode the hNIS gene. Timing of cellular uptake of radioiodide ^131^I in human pancreatic carcinoma cells PANC-1 was assessed using radiouptake assays. Viral biodistribution was determined in nude mice bearing PANC-1 xenografts, and infection in tumors confirmed histologically and optically via Green Fluorescent Protein (GFP) and bioluminescence. Timing characteristics of enhanced radiouptake in xenografts were assessed via ^124^I-positron emission tomography (PET). Detection of systemic administration of virus was investigated with both ^124^I-PET and 99m-technecium gamma-scintigraphy.

**Results:**

GLV-1h153 successfully facilitated time-dependent intracellular uptake of ^131^I in PANC-1 cells with a maximum uptake at 24 hours postinfection (P<0.05). *In vivo*, biodistribution profiles revealed persistence of virus in tumors 5 weeks postinjection at 10^9^ plaque-forming unit (PFU)/gm tissue, with the virus mainly cleared from all other major organs. Tumor infection by GLV-1h153 was confirmed via optical imaging and histology. GLV-1h153 facilitated imaging virus replication in tumors via PET even at 8 hours post radiotracer injection, with a mean %ID/gm of 3.82±0.46 (P<0.05) 2 days after intratumoral administration of virus, confirmed via tissue radiouptake assays. One week post systemic administration, GLV-1h153-infected tumors were detected via ^124^I-PET and 99m-technecium-scintigraphy.

**Conclusion:**

GLV-1h153 is a promising oncolytic agent against pancreatic cancer with a promising biosafety profile. GLV-1h153 facilitated time-dependent hNIS-specific radiouptake in pancreatic cancer cells, facilitating detection by PET with both intratumoral and systemic administration. Therefore, GLV-1h153 is a promising candidate for the noninvasive imaging of virotherapy and warrants further study into longterm monitoring of virotherapy and potential radiocombination therapies with this treatment and imaging modality.

## Introduction

Oncolytic viral therapies have shown such promise in preclinical trials as a novel cancer treatment modality, that several phase I and II trials are already underway [1]. Oncolytic vaccinia virus strains have been of particular interest due to several advantages. Vaccinia's large 192-kb genome [2] enables a large amount of foreign DNA to be incorporated without significantly reducing the replication efficiency of the virus, which has been shown to be the case with some adenoviruses [3]. It has fast and efficient replication, and cytoplasmic replication of the virus lessens the chance of recombination or integration of viral DNA into cells [3], [4]. Vaccinia has also been shown capable of immune evasion and of infecting a wide variety of cells [4], [5], [6], [7], [8], [9], [10], [11], [12], 13. Perhaps most importantly, its safety profile after its use as a live vaccine in the WHO's smallpox vaccination makes it particularly attractive as an oncolytic agent and gene vector [14]. Furthering its safety profile, vaccinia immunoglobulin and antiviral drugs are available if needed [15].

We have previously reported on the construction and generation of a novel attenuated replication-competent vaccinia virus (VACV), GLV-1h153, a derivative of parental virus GLV-1h68 engineered to carry the human sodium iodide symporter (hNIS) [16]. hNIS, an intrinsic plasma membrane protein, facilitates transport of several carrier-free radiotracers such as radioiodine and technecium-pertechnetate (^99m^TcO_4_) [17]. GLV-1h153 facilitated enhanced dose-dependent radiouptake in cell culture and effective replication and killing of pancreatic cancer cells both in cell culture and in animal models, without hindering replication or oncolytic capability. Furthermore, GLV-1h153 facilitated enhanced radiouptake in tumors which was readily detected by positron emission tomography (PET), a deep tissue imaging modality. The noninvasive tracking of virus delivery may offer clinicians the ability to correlate efficacy and therapy, monitor potential viral toxicity, and provide a more sensitive and specific diagnostic technique to detect tumor origin and, more importantly, presence of metastases [18], [19].

In this study, we aimed to further this work by determining the tissue distribution and spread of GLV-1h153, and explore the timing dynamics between viral infection, uptake of radioiodine, and oncolysis. The optical, histologic, and deep tissue viral detection capability was investigated. Moreover, we determined the radioiodine retention capacity of tumors transduced with hNIS via GLV-1h153, and whether PET imaging can be accurately quantitative. Finally, we investigate the potential to detect virus replication in tumors treated systemically via both ^124^I-PET and ^99m^TcO_4_-mediated gamma-scintigraphy.

## Results

### GLV-1h153 infected PANC-1 cells demonstrated time-dependent enhanced uptake of carrier-free radioiodide

**Figure 1:**
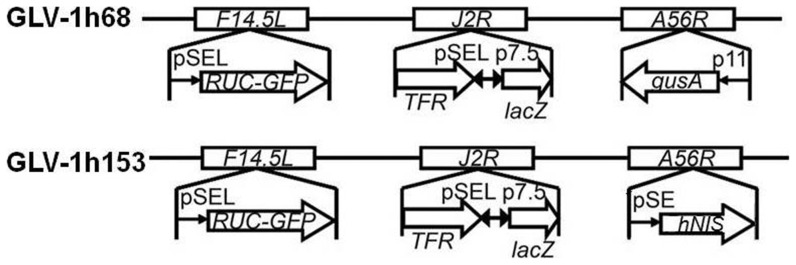
GLV-1h153 construct. GLV-1h153 was derived from GLV-1h68 by replacing the *gusA* expression cassette at the *A56R* locus with the *hNIS* expression cassette through homologous recombination. Both viruses contain RUC-GFP and *lacZ* expression cassettes at the *F14.5L* and *J2R* loci, respectively. PE, PE/L, P11, and P7.5 are VACV synthetic early, synthetic early/late, 11K, and 7.5K promoters, respectively. TFR is human transferrin receptor inserted in the reverse orientation with respect to the promoter PE/L.

**Figure 2:**
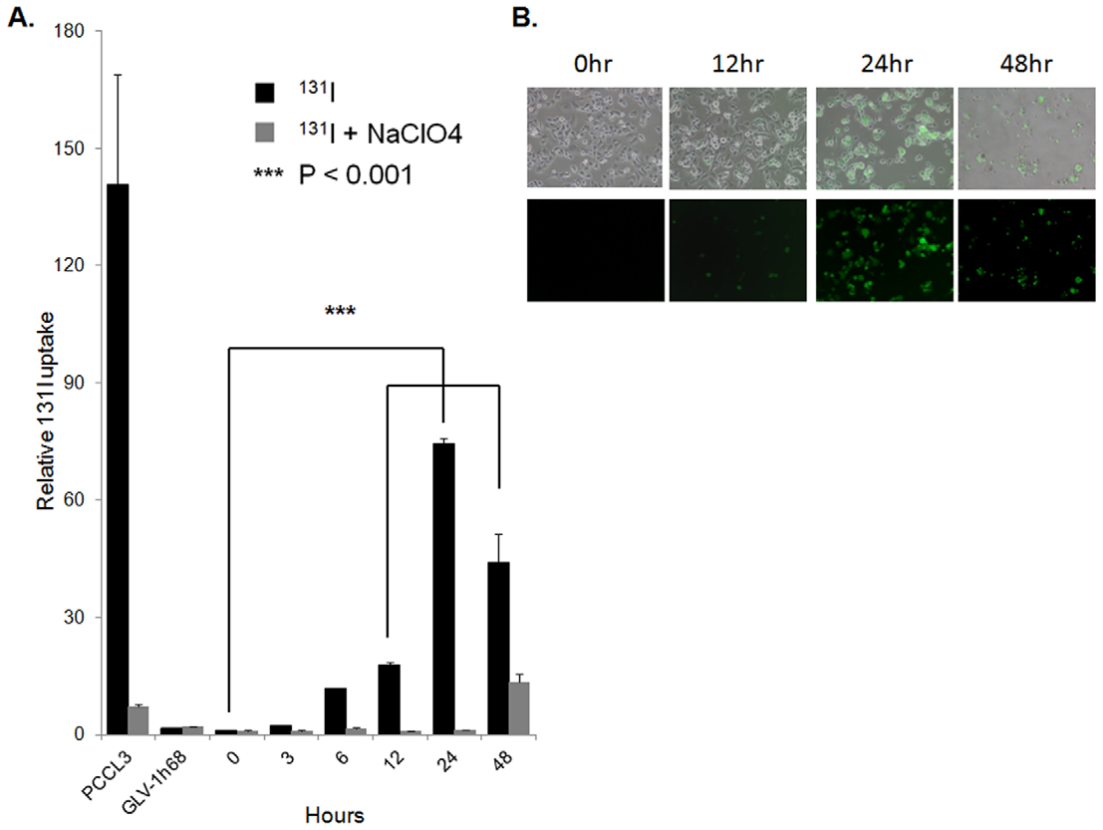
Assessment of ^131^I radiouptake of GLV-1h153-infected PANC-1 cells in culture. A. PANC-1 cells were mock infected or infected with an MOI of 1.0 of GLV-1h153 or GLV-1h68. PCCL3 was used as a positive control. Radiouptake was time-dependent with a maximal uptake of over 70 fold as compared to control at 24 hrs after infection. When GLV-1h153-infected cells were treated with competitive inhibitor of hNIS, NaClO_4_, radiouptake within cells decreased as compared to untreated cells. B. Maximum GFP expression expression with an MOI of 1.0 was also at 24 hrs after infection, with a decrease of both radiouptake and GFP expression by 48 hrs.

GLV-1h153 was derived from parental virus GLV-1h168 as previously described (Figure 1) [16]. To establish that hNIS-mediated radiouptake was time-dependent, cells were mock infected or infected at an MOI of 1.0 with GLV-1h153 or GLV-1h68, then treated with ^131^I at various times after infection. Normal rat thyroid cell line PCCL3 was used as a positive control. PANC-1 cells infected with GLV-1h153 showed a time-dependent radiouptake, with >70 fold increased maximum uptake as compared to negative control at 24 hours after infection (P<0.001) (Figure 2A). This maximum radiouptake time point correlated with peak GFP expression (Figure 2B). Moreover, when cells were treated with sodium perchlorate (NaClO_4_), a competitive inhibitor of hNIS, radiouptake decreased in GLV-1h153-treated cells, indicating hNIS-specific radiouptake. By 48 hours after infection, a decrease of both radiouptake and GFP expression is seen secondary to the oncolytic effects of the virus.

### GLV-1h153 persisted in tumor xenografts with clearance from almost all other organs by 5 weeks post injection

**Figure 3:**
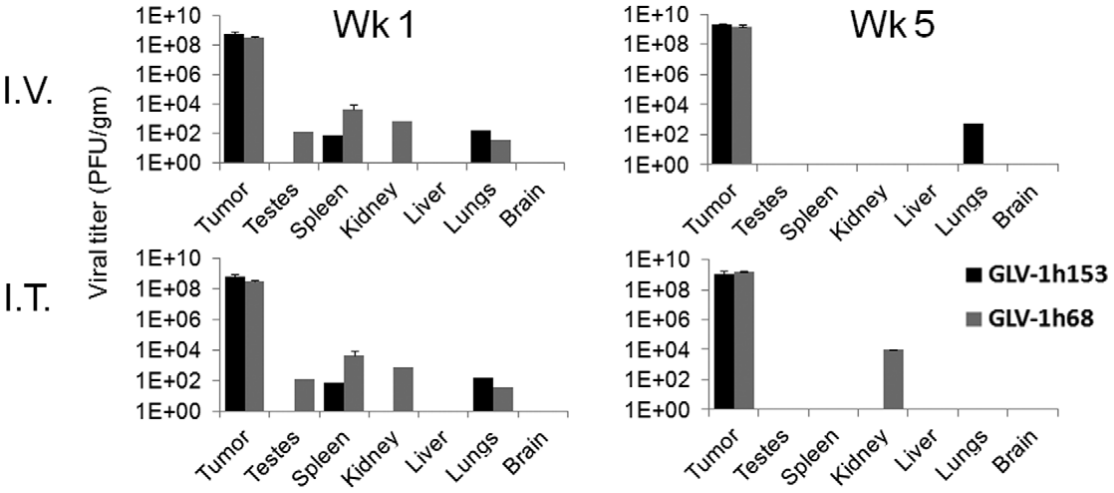
Viral biodistribution of GLV-1h153 in animal models. GLV-1h153 and GLV-1h68 particles were recovered from tumor tissues of virus-treated animals at 1 and 5 weeks after viral injection in the order of 10^9^ viral particles in both the IT and IV group (3–4 mice per group per time point). Only trace amounts of virus were detected in the testes, spleen, kidney and lungs for both viruses and for both the IV and IT group at 1 week. By 5 weeks, virus replication persisted at almost 10^9^ PFU per gram of tissue in the tumors, while residual viral particles were cleared in most organs.

To assess viral biodistribution *in vivo*, animals were treated either intratumorally (ITly) or intravenously (IV) with GLV-1h153 or parent virus GLV-1h68. Tumor and organs were then harvested 1 and 5 weeks post virus injection. GLV-1h153 viral particles were recovered from tumor tissue of virus-treated animals at 1 and 5 weeks post injection of virus, in the order of 10^9^ plaque forming units (PFU) in both the IT and IV groups. Only trace amounts of virus were detected in the spleen and lungs for the IT and IV groups at 1 week. By 5 weeks post virus administration, virus particles persisted at almost 10^9^ PFU/gram of tissue in the tumors, while the virus was mostly cleared in other organs, with residual viral particles remaining only in lung in the IV group, and kidney in the IT group (Figure 3). Results were similar with the GLV-1h68 treated group, suggesting that insertion of the hNIS gene did not alter replication capacity or spread of GLV-1h153. Average weights of tumors per group used for biodistribution studies ranged from 423 gm to 573 gm at 1 week post virus injection, and 222–577 gm at 5 weeks post virus treatment.

### Histology and optical imaging confirmation of GLV-1h153 infection of pancreatic tumor xenografts

**Figure 4:**
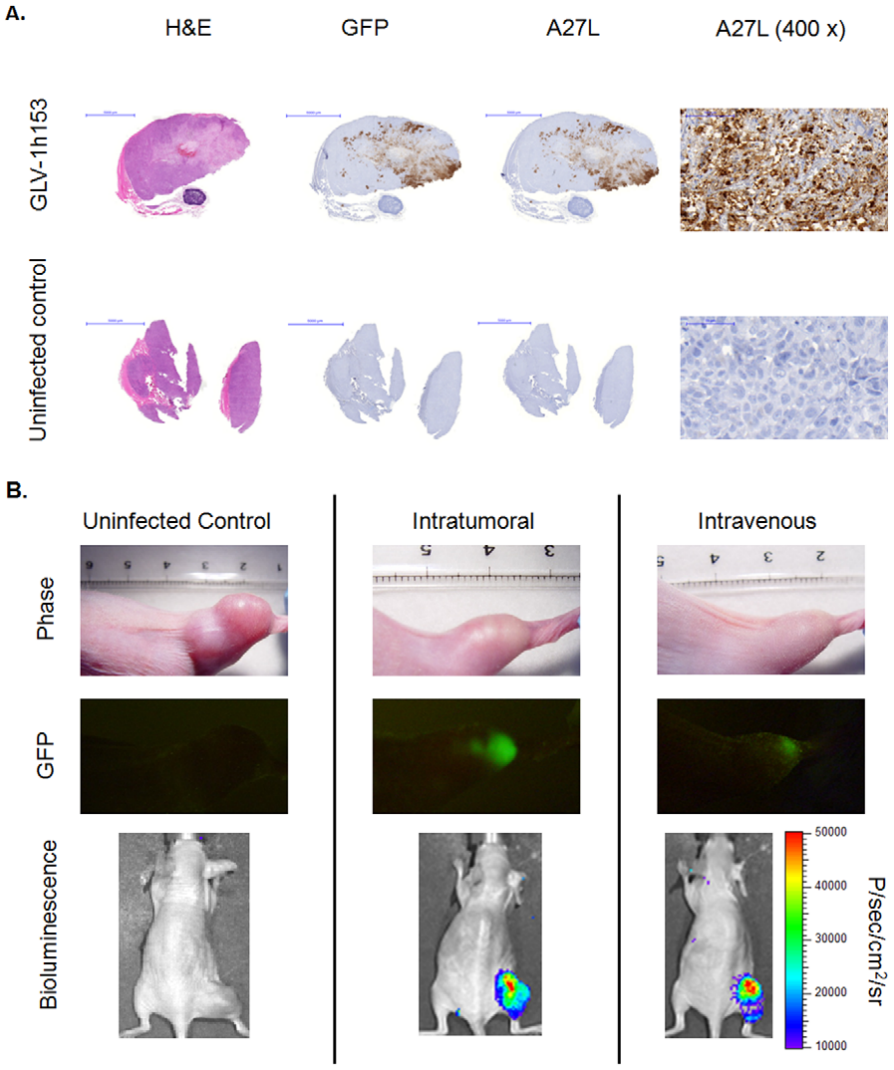
Optical and histologic detection of viral replication using vaccinia marker genes. A. Presence of GLV-1h153 in tumors was detected histologically, shown here with the IT group 2 days post virus injection. Areas staining positive with antibodies against GFP and VACV antigen A27L corresponded and were easily visualized in GLV-1h153-injected tumors, whereas no staining was evident in untreated tumors. Areas of A27L staining is also shown at 400× magnification. B. GFP expression was optically monitored, shown here 5 weeks postinjection. Control tumors were larger than GLV-1h153-treated tumors, both when administered intratumorally or intravenously. GLV-1h153 was also detected via bioluminescence imaging 2 weeks posttreatment.

To confirm virus presence in tumors and correlate this with PET imaging of tumors 2 days after treatment, 2 animals injected ITly with GLV-1h153 or GLV-1h168, as well as PBS controls, were sacrificed 2 days posttreatment. All tumors treated with either GLV-1h153 or GLV-1h68 stained positive for vaccinia A27L antigen, yielding a brownish precipitate compared to the blue-purple hematoxylin background seen with uninfected areas and control tumors. Furthermore, tumor areas staining for GFP corresponded to areas positive for A27L (Figure 4A). Virus presence in tumors could also be detected via optical imaging of GFP, which persisted at even 5 weeks post injection, with tumor regression evident as compared to control. Furthermore, virus was detectable in virus-infected tumors by bioluminescence imaging 2 weeks after viral administration (Figure 4B).

### GLV-1h153-enhanced radiouptake in intratumorally infected PANC-1 tumor xenografts was readily imaged via PET and mediated radiotracer retention

**Figure 5:**
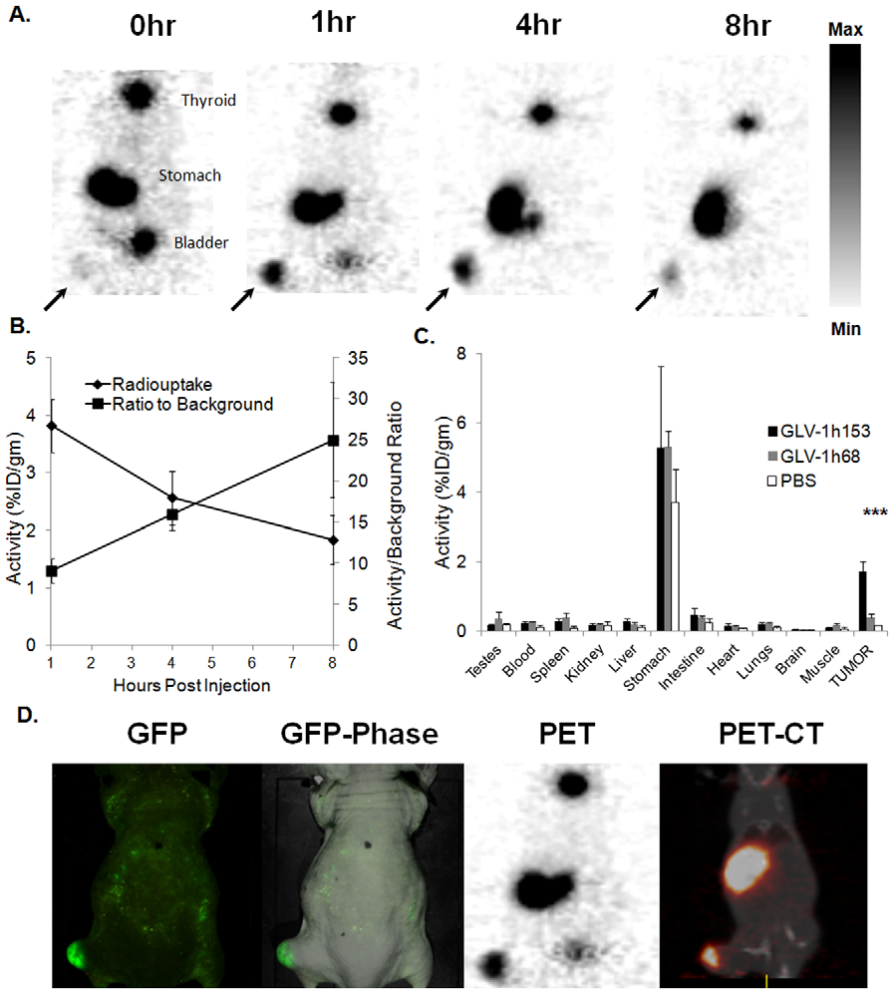
PET-detection of timing characteristics of GLV-1h153-facilitated radiouptake by intratumorally-treated PANC-1 xenografts. A. Two ×10^7^ PFU of GLV-1h153,was injected ITly into PANC-1 hindleg tumor-bearing mice (3 mice). ^124^I PET scanning was obtained 48 hours after infection and 1 hour after radiotracer administration. GLV-1h153-infected PANC-1 tumors were easily visualized (shown with arrows). Stomach and thyroid were also visualized due to native NIS expression, and the bladder due to tracer excretion. Gray scale reflects maximum and minimum radiouptake within the tumor regions on PET images, with greater black intensity signifying greater radiouptake. B. Although absolute radiouptake decreased between 1 and 8 hrs after radiotracer administration, ratio of uptake versus background steadily increased. C. Enhanced radiouptake in tumors infected with GLV-1h153 compared to GLV-1h68 and PBS 8 hrs after radiotracer injection was confirmed via tissue radiouptake assays and correlated with quantitative PET (2–3 mice per group). Enhanced uptake in the stomach is evident in all groups due to native NIS expression. d. Tumor infection with GLV-1h153 was confirmed with GFP and CT-PET in the same mice 2 days post intratumoral injection.

After successful cell culture radioactivity uptake studies, the feasibility of utilizing GLV-1h153 in combination with carrier-free ^124^I radiotracer to image infected PANC-1 tumors was then explored, in addition to the timing dynamics of virus infection and radiouptake. hNIS protein expression in the PANC-1 tumor-bearing animals after GLV-1h153 administration was visualized by ^124^I-PET. Carrier free ^124^I was IVly administered 48 hours after IT virus injection and PET imaging performed 1, 2, and 8 hours after radiotracer administration. Tumor radioactivity values (%ID/g) were measured and compared to background utilizing four averaged ROIs. The maximal average levels of radioactivity in GLV-1h153-injected tumors were 3.82±0.46%ID/gm 1 hour after radiotracer administration, whereas the PBS-injected tumors could not be visualized, and therefore were not significantly above background (P<0.001). When imaged serially over 1, 2, and 8 hours, absolute activity in tumors declined; however, the ratio of activity to background increased from 9.11±1.48 (P<0.001) to 25.0±7.05 (P<0.01) (Figure 5A and 5B). Enhanced radiouptake in GLV-1h153 injected tumors compared to 1) other organs and 2) GLV-1h68- and PBS-injected tumors was confirmed in these mice via radiouptake assay at 8 hours post radiotracer administration, and the activity in GLV-1h153-infected tumors correlated well with PET activity, at 1.71±0.30 (P<0.001 compared to both PBS and GLV-1h68 groups) and 1.84±0.42 (P<0.01 also compared to both PBS and GLV-1h68), respectively, whereas uptake in control tumors were again not above background (Figure 5C). Weights of the imaged tumors used for tissue radiouptake assays averaged 554 gm. The presence of the virus in tumors was confirmed in these particular mice via GFP optical imaging, and the enhanced localized activity in the tumors confirmed via fusing CT scans with PET imaging (Figure 5D).

### GLV-1h153-enhanced radiouptake in intratumorally infected PANC-1 tumor xenografts was readily imaged via PET

**Figure 6:**
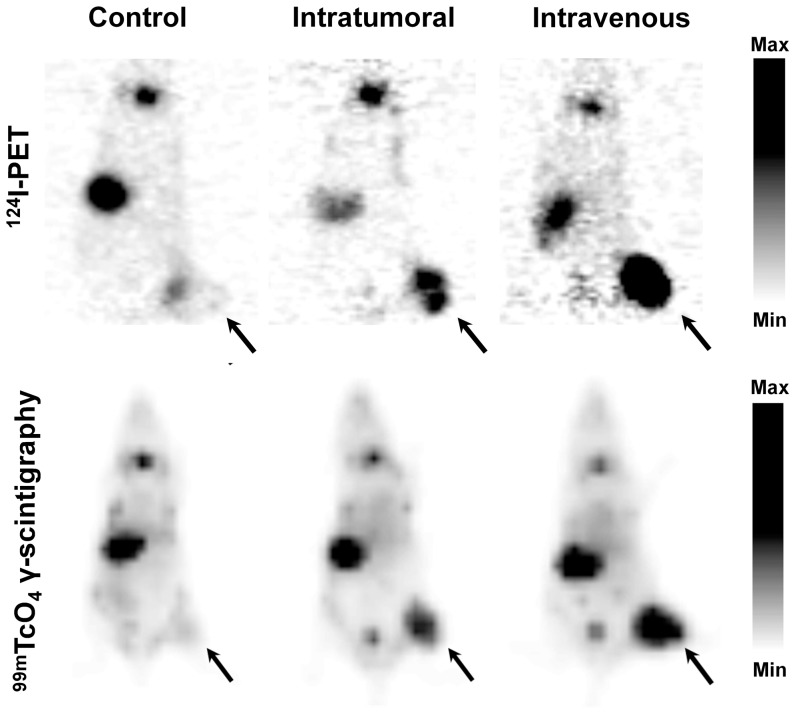
PET imaging and γ-scintigraphy of GLV-1h153 replication in intravenously treated PANC-1 xenografts. Three groups of 2 animals each, bearing subcutaneous PANC-1 xenografts on the right hindleg, were injected IVly (2 mice) or ITly (2 mice) with 2×107 PFU of GLV-1h153, or PBS (2 mice). One mouse from each group was imaged with ^124^I-mediated PET scanning and the other imaged with ^99m^TcO_4_-mediated γ-scintigraphy. GLV-1h153 replication in both ITly- and IVly-treated PANC-1 tumors were easily visualized with both modalities (shown with arrows). Stomach and thyroid were also visualized due to native NIS expression, and the bladder due to tracer excretion. Gray scale reflects maximum and minimum radiouptake within the tumor regions by each modality, with greater black intensity signifying greater radiouptake.

To determine if GLV-1h153 mediated hNIS transfer can also be detected with systemic delivery of virus and with ^99m^TcO_4_-mediated γ-scintigraphy, 3 groups of 2 animals each, bearing subcutaneous PANC-1 xenografts on the right hindleg, were injected IVly (2 mice) or ITly (2 mice) with 2×10^7^ PFU of GLV-1h153, or PBS (2 mice). One mouse from each group was imaged with ^124^I-mediated PET scanning and the other imaged with ^99m^TcO_4_-mediated γ-scanning. Viral mediated uptake was successfully detected and easily visualized with ^99m^TcO_4_, (Figure 6). Like the PET images, uptake was also noted in the bladder due to radiotracer excretion, as well as the thyroid and stomach due to intrinsic hNIS expression.

## Discussion

Pancreatic cancer in is the fourth leading cause of cancer death in the United States [20], and objective response to single agent or combination chemotherapies occurs in less than 20% of patients which is never curative [21]. These results explain the active investigation underway seeking novel therapies, which may also work synergistically in combination with conventional treatment options, for this disease.

Oncolytic viral therapy is emerging as a novel cancer treatment. Engineered VACVs have been successfully used as direct oncolytic agents, capable of preferentially infecting, replicating within, and killing a wide variety of cancer cell types [16], [22]. One such promising virus strain is GLV-1h68, which has shown efficacy in the treatment of several human cancers in preclinical models, and is currently being tested in phase I human trials [1]. Vaccinia's large insertional cloning capacity allows for the inclusion of several functional and therapeutic transgenes [2]. With the insertion of reporter genes not expressed in uninfected cells, viruses can be localized and the course of viral therapy can be monitored in patients. Future clinical studies with VACV may benefit from the ability to noninvasively and serially identify sites of viral targeting and to measure the level of viral infection for correlation with safety, efficacy, and toxicity [18], [19], [23]. Such real-time tracking would also provide useful viral dose and administration schedule information for optimization of therapy and would obviate the need for multiple and repeated tissue biopsies.

We have previously reported on the generation of a novel recombinant VACV, GLV-1h153, derived from GLV-1h68, which has been engineered for specific targeted treatment of cancer and the additional capability of facilitating noninvasive imaging of viral replication in tumors. To our knowledge, GLV-1h153 is the first oncolytic vaccinia virus expressing the hNIS protein that can efficiently infect tumors and simultaneously facilitate deep tissue imaging of viral replication. hNIS is an intrinsic plasma membrane protein which mediates the active transport and concentration of iodide in the thyroid gland cells and some extra-thyroidal tissues [24], [25], [26], [27]. It is also one of several human reporter genes currently being used in preclinical studies and has been used in clinical studies imaging adenoviral-mediated hNIS transfer in prostate cancer [1], [28]. hNIS gene transfer via viral vector may allow infected tumor cells to concentrate several carrier-free radionuclide probes such as ^123^I, ^124^I, ^125^I, and ^99m^TcO_4_,which have long been approved for human use, facilitating for both deep tissue imaging of viral therapy and potential targeted radiotherapy [17].

Oncolytic viruses encoding hNIS that have been investigated to date include several adenoviruses [29], [30], [31] and measles viruses [32], [33], [34], [35], [36], [37], [38], as well as a vesicular stomatitis virus (VSV) [39]. Results have been promising; however, there are several disadvantages to each strain. Although adenoviruses can infect a broad spectrum of cells with high infection efficiency, lack of efficient viral replication and tumor transduction capacity especially after foreign gene insertion have limited their clinical application [29], [30], [31]. Similarly, measles viruses have also been reported to have inefficient transduction within tumors, when investigations of possible combination therapy of MV-*NIS* and radioiodine failed to produce synergistic therapeutic effects [38]. The VSV encoding hNIS also showed promise, however, both VSV and measles are RNA viruses which may increase their chances of integration into the host genome [3]. VACV's safety profile is unsurpassed, and its high replication capacity and efficient cell to cell spread may enable it to overcome many of the limitations associated with other oncolytic viral vectors.

In this study, we assessed the imaging and timing characteristics, and tissue distribution and spread of GLV-1h153. In order to determine how the dynamic state between viral infection, replication, and lysis of tumor cells may affect GLV-1h153-mediated cellular radiouptake, time-dependent radiouptake assays were performed first in cell culture. GLV-1h153-mediated expression of the hNIS protein in infected PANC-1 cells in culture resulted in time-dependent and hNIS-specific uptake of the radiotracer ^131^Iwith radiouptake reaching the maximum at 24 hpi (∼70-fold above control) and starting to decrease at 48 hpi due to the oncolytic effects of the virus. The hNIS protein may not have been translocated and inserted into the cell membrane to form a functional transporter during the initial 12- to 24-hour period after infection. During the late, prelytic phase of viral infection (72 hours and beyond), the hNIS transporter could be impaired, and following cell oncolysis, the accumulated ^131^I radiotracer would be lost. Thus, there seems to be a relatively narrow window, ∼24 to 48 hours after viral infection of PANC-1 cells, during which the hNIS reporter is maximally functional. These results were similar to those seen with the hNET-expressing vaccinia virus GLV-1h99 [40].

We then explored the biodistribution of GLV-1h153 *in vivo*, which are likely to affect imaging characteristics especially with systemic administration of the virus. Viral biodistribution assays revealed preferential replication and persistence of viral particles in tumors at even 5 weeks after both intratumoral and intravenous viral administration at ∼10^9^ PFU per gram tissue, with most of the virus cleared from all other organs and trace residual amounts found in the lung and kidney. This highlights GLV-1h153's promising safety profile, inherent affinity of the viruses to tumor cells, and the “tumor homing" capability of systemic administration of the virus. This is of particular interest and importance for clinical applicability and treatment of widespread disease..

Mechanisms of vaccinia viral oncogenesis remain speculative and unproven. All cell lines, healthy and tumorous, take up the vaccinia virus particles presumably by macropinocytosis [41]. Cytoplasmically replicating wild-type and mutant VACVs including GLV-1h153 show tumor-specific entry and replication upon systemic delivery in several tumor types from different species, including mouse and canine [42], [43]. It has been shown that oncolytic viruses target cancers that overexpress proteins such as ribonucleotide reductase, DNA repair enzymes, and proteins rendering them resistant to apoptosis, characteristics that tend to make tumor cells resistant to chemotherapy and radiation therapy [44]. With regards to GLV-1h153 in particular, deletional mutations introduced into nonessential VACV genes such as J2R (encoding thymidine kinase (TK)) and the vaccinia growth factor (VGF) gene were shown to produce significant attenuation of the wt Western Reserve (WR) strain, further enhancing tumor targeting [5]. Parent virus GLV-1h68 has already been tested in humans in a phase I clinical trial, with no adverse events or systemic toxicity noted in doses up to 3×10E9 PFU (2011 ASCO meeting poster) [1]. Furthermore, when GLV-1h68 was used against both human melanoma and syngeneic melanoma xenografts in mice, preferential replication of virus in tumors was demonstrated in both showing that it is unlikely that a host limitation of vaccinia in mouse tissue compared to humans is the explanation for the preferential selection [8]. Further investigation is needed to elucidate the exact mechanisms rendering vaccinia viruses highly selective and oncogenic in tumors.

We then assessed the feasibility, timing characteristics, and potential for radioiodine retention by imaging GLV-1h153-infected tumors. Mice were treated intratumorally with GLV-1h153, non-hNIS expressing parent virus GLV-1h68, and PBS, and imaged 48 hours after with carrier free ^124^I. The quantitative ^124^I -PET showed that imaging of GLV-1h153 infection of PANC-1 tumors is feasible after direct tumor injection. To confirm presence of virus in treated tumors, histologic staining was performed against both GFP and VACV A27L antigen 2 days after treatment and was positive for viral infection. Further, GLV-1h153-treated tumors were optically imaged via GFP and bioluminescence. Multimodal detection of viral treatments would be undoubtedly useful for the tracking and monitoring of viral therapy in clinical trials.

The timing of PET imaging after ^124^I administration was also shown to be important, as radioactivity levels (% ID/gm) in GLV-1h153-infected tumors was highest during the first 1-hour period after tracer administration. This difference is likely the effect of radioefflux from cells, however, the tumor uptake to background ratio actually increased in tumors with time, thus adequate retention of radioiodine is mediated even 8 hours after radiotracer injection. This was also seen with other hNIS-encoding viruses [31], [32], [33]. Retention of radioiodine within tumors is promising for possible targeted combination therapy with systemically administered therapeutic radioiodine such as ^131^I, as dose effects of radiotherapy increase with longer retention times [45], [46]. Enhanced radiouptake in GLV-1h153-injected tumors compared to other organs, as well as GLV-1h68- and PBS-injected tumors was confirmed in these mice via tissue radiouptake assay at 8 hours post radiotracer administration, and correlated well with quantitative PET.

Finally, we investigated whether systemically-injected GLV-1h153, which was seen to preferentially accumulate in tumors in our bioditribution assays, could in turn be detected with PET. Further, we ascertained if this hNIS vector can facilitate enhanced uptake of both radioiodine and technecium pertechtenate. Mice were treated intratumorally or systemically with GLV-1h153 or PBS, and imaged 1 week after with carrier free ^124^I or ^99m^TcO_4_. Our results show that systemically administered GLV-1h153 can be successfully detected by both deep tissue imaging modalities. The implications of this are two-fold: first, detection of systemically administered treatment widens the clinical applicability of this imaging system for the treatment of widespread disease and noninvasive monitoring of viral therapy; second, when image resolution is not of particular importance, detection with gamma scintigraphy, a less expensive and more widely available imaging modality as compared to PET, is feasible [47], [48].

Therefore, this study has furthered understanding of the biodistribution and tumor-specific imaging characteristics following treatment of pancreatic tumor xenografts with GLV-1h153, a novel VACV expressing the *hNIS* reporter gene. Both intratumorally and intravenously infected pancreatic tumors were readily imaged with the clinically approved radiopharmaceuticals and imaging modalities of ^124^I-PET and ^99m^TcO_4_-mediated gamma-scintigraphy. Moreover, intratumoral GLV-1h153 infection of tumor xenografts facilitated radioiodine dose accumulation and retention at even 8 hours post radioiodine injection. These findings warrant further investigation into possible long term monitoring of viral therapy, as well as synergistic or additive effects of systemically administered radiotherapy combined with this novel treatment and imaging modality.

## Materials and Methods

### Virus and cell culture

African green monkey kidney fibroblast CV-1 cells and human pancreatic ductal carcinoma PANC-1 cells were purchased from American Type Culture Collection (ATCC) (Manassas, VA) and were grown in Dulbecco's modified Eagle's medium (DMEM) supplemented with 1% antibiotic-antimycotic solution (Mediatech, Inc., Herndon, VA) and 10% fetal bovine serum (FBS) (Mediatech, Inc.) at 37°C under 5% CO_2_. Normal rat thyroid follicular cells, PCCL3 (a gift from Dr. J. Fagin, MSKCC) were maintained in Coon's modified medium (Sigma, St. Louis, MO), 5% calf serum, 2 mM glutamine, 1% penicillin/streptomycin, 10 mM NaHCO3, and 6H hormone (1 mU/ml bovine TSH, 10 ug/ml bovine insulin, 10 nM hydrocortisone, 5 ug/ml transferrin, 10 ng/ml somatostatin, and 2 ng/ml L-glycyl-histidyl-lysine) at 37°C under 5% CO_2_. GLV-1h68 was derived from VACV LIVP, as described previously [5]. GLV-1h153 was derived from GLV-1h68, as also previously described [16].

### Cell culture radiouptake assay

Radiouptake in cells infected with GLV-1h153 was compared to rat thyroid cell line endogenously expressing NIS (PCCL3), and to cells infected with parental virus GLV-1h68 or mock infected. Cells were plated at 5×10^5^ cells per well in 6-well plates. Twenty-four hours after infection, cells were treated with 0.5 µCi of either carrier free ^131^I or ^131^I with 1 mM of sodium perchlorate (NaClO_4_), a competitive inhibitor of hNIS for a 60-minute incubation period. Media was supplemented with 10 µM of sodium iodide (NaI). Iodide uptake was terminated by removing the medium and washing cells twice with PBS. Finally, cells were solubilized in lysis buffer for residual radioactivity, and the cell pellet-to-medium activity ratio (cpm/g of pellet/cpm/mL of medium) calculated from the radioactivity measurements assayed in a Packard γ-counter (Perkin Elmer, Waltham, MA). Results were expressed as change in uptake relative to negative uninfected control. All samples were done in triplicate.

### Viral biodistribution studies in animal models

All animal studies were performed in compliance with all applicable policies, procedures, and regulatory requirements of the Institutional Animal Care and Use Committee, the Research Animal Resource Center of Memorial Sloan-Kettering Cancer Center, and the National Institutes of Health “Guide for the Care and Use of Laboratory Animals. PANC-1 xenografts were developed in 6- to 8-week-old male nude mice (NCI:Hsd:Athymic Nude-*Foxn1*nu, Harlan) by implanting 2×10^6^ PANC-1 cells in PBS subcutaneously in the left hindleg. Tumor growth was recorded once a week in 3 dimensions using a digital caliper and reported in mm^3^ using the formula (length×width×[height-5]). When tumors reached 100–300 mm^3^, mice were injected intratumorally (ITly) or intravenously (IVly) via the tail vein with a single dose of 2×10^6^ PFUs GLV-1h153 or GLV-1h68 in 100 µL PBS. Animals were observed daily for any sign of toxicity, and body weight checked weekly. Tissue from organs - lung, liver, spleen, kidney, brain, testes - as well as from tumor, were harvested at 1 and 5 weeks postinjection of virus, weighed, suspended in 500 mL PBS containing protease inhibitor, and homogenized for 30 seconds at a speed of 6500 rpm. Three to 4 mice were used per group. After homogenization, samples were subjected to 3 freeze–thaw cycles. Samples were then centrifuged for 5 minutes at 3000 *g* at 4°C, supernatants collected, and serial dilutions made. Standard plaque assays were performed on 24-well plates of confluent CV-1 cells, with all samples assessed in duplicate.

### Histologic confirmation of GLV-1h153 infection of pancreatic tumor xenografts

At 2 days post IT viral injection, animals were sacrificed and the tumors harvested. Tissue sections were deparaffinized by serial passages in xylene, then subjected to a graded series of ethanol washes before endogenous peroxidase activity was blocked by incubation in a 50% by volume solution of 3% H_2_O_2_/methanol for 10 minutes. The immunhistochemistry detection of both anti- GFP and anti-VACV A27L antigen antibodies was performed at the Molecular Cytology Core Facility of Memorial Sloan Kettering Cancer Center using Discovery XT processor (Ventana Medical Systems). The chicken polyclonal anti-GFP antibody (Abcam, ab13970) was used in a 2 ug/mL concentration. Preceding the primary antibody incubation, the tissue sections were blocked for 30 min in 10% normal goat serum, 2% BSA in PBS. The incubation with the primary antibody was done for 3 hours, followed by 60 minutes incubation with biotinylated goat anti-chicken IgG (Vector labs, T1008) in 7.5 ug/mL concentration. For the VACV A27L antigen detection, slides were incubated with polyclonal antibody produced in rabbits against synthetic peptide AKKIDVQTGRRPYE (the C-terminal of A27L vaccinia protein) (custom made by GenScript Corporation, Piscataway, NJ) at a dilution of 1∶1000 for 5 hours, followed by 60 minutes incubation with biotinylated goat anti-rabbit IgG (Vector labs, PK6101) in 1∶200 dilution. Secondary Antibody Blocker, Blocker D, Streptavidin- HRP and Diaminobenzidine DAB detection kit (Ventana Medical Systems) were used according to the manufacturer instructions. Slides were then couterstained with Hematoxylin (Ventana Medical Systems, 760–2021) and Bluing Reagent (Ventana Medical System, 760–2037) 4 minutes before mounting also according to the manufacturer instructions. Negative IgG controls were used for both antibodies for comparison.

### Optical imaging of pancreatic tumor xenografts

GFP expression of tumors infected with GLV-1h153 was visualized directly using UV light fluorescence. For the bioluminescence imaging, animals were analyzed for the presence of virus-dependent luciferase activity 2 weeks post virus injection. For this purpose, mice were injected intraperitoneally with a mixture of 5 µL coelenterazine (Sigma; 0.5 mg/mL diluted ethanol solution) and 95 µL of luciferase assay buffer (0.5 M NaCl, 1 mM EDTA, and 0.1 M potassium phosphate, pH 7.4). Bioluminescence was then measured with an IVIS 100 Imaging system (Xenogen) by collecting photons for 1 minute from dorsal views of the animals and analyzed using the LIVINGIMAGE 2.5 software (Xenogen).

### PET imaging of intratumorally treated animal models

Three groups of 2–3 animals bearing subcutaneous PANC-1 xenografts on the left hindleg were injected ITly with 2×10^7^ PFU GLV-1h153 (3 mice), 2×10^7^ PFU GLV-1h68 (2 mice), or PBS (2 mice). Two days after viral injection, 140 µCi of ^124^I was administered via the tail vein. At hours 1, 2, and 8 after radiotracer administration, 3-dimensional list-mode data were acquired using an energy window of 350 to 700 keV, and a coincidence timing window of 6 nanoseconds. Imaging was performed using a Focus 120 microPET dedicated small animal PET scanner (Concorde Microsystems Inc, Knoxville, TN). These data were then sorted into 2-dimensional histograms by Fourier rebinning. The image data were corrected for (a) nonuniformity of scanner response using a uniform cylinder source-based normalization, (b) dead time count losses using a single-count rate-based global correction, (c) physical decay to the time of injection, and (d) the ^124^I branching ratio. The count rates in the reconstructed images were converted to activity concentration (%ID/g) using a system calibration factor (MBq/mL per cps/voxel) derived from imaging of a mouse-size phantom filled with a uniform aqueous solution of ^18^F. Image analysis was performed using ASIPro (Siemens Pre-clinical Solutions, Knoxville, TN). Four regions of interest (ROIs) were manually drawn on each tumor and averaged for comparison, with the mean injected value ± SD recorded.

### Tissue radiouptake assay

Two days following IT injection of either GLV-1h153, GLV-1h68, or PBS, mice were injected via tail vein with 140 µCi of ^124^I (2–3 mice per group). At 8 hours post radiotracer injection and PET imaging, organs including heart, lung, liver, spleen, kidney, thyroid, muscle, blood and tumors were collected, weighed, and their radioactivity determined with a γ-counter (Perkin Elmer, Waltham, MA). Data was normalized as above and also expressed %ID/g.

### PET imaging and gamma-scintigraphy of intravenously treated animals

To investigate if intravenously injected GLV-1h153 replication can be successfully detected within PANC-1 tumor xenografts, 3 groups of 2 animals each, bearing subcutaneous PANC-1 xenografts on the right hindleg, were injected IVly (2 mice) or ITly (2 mice) with 2×10^7^ PFU of GLV-1h153, or PBS (2 mice). One mouse from each group was imaged 1 week after systemic virus administration with ^124^I-mediated PET scanning as above, and the other imaged with ^99m^TcO_4_-mediated gamma-scintigraphy 1 hr post ^99m^TcO_4_ radiotracer administration. The one week time point was chosen for correlation with systemic tissue biodistribution studies, with knowledge that systemic toxicity is unlikely to develop within the first few hours or days after treatment. Further, studies using bioluminescence imaging 2 days after systemic administration of the virus were unable to detect virus colonization of organs or tumor likely due to the initial low level of replication of the virus. Ventral and dorsal planar images of the *in vivo* distribution of ^99m^TcO_4_ were simultaneously acquired using the dual-detector gamma camera sub-system of the XSPECT small-animal SPECT-CT system (Gamma Medica, Northridge, CA). The detectors were fitted with low-energy, high-resolution parallel-hole collimators and images acquired over 10 minutes using a 140 keV±10% ^99m^TcO_4_ photopeak energy window and a 56×56 image matrix (pixel size: 2.2×2.2 mm). Images were corrected for non-uniformity of response using a measured “sensitivity map"; no attenuation or scatter correction was applied. The resulting images were parameterized in terms of the percent of the %ID/gm, corrected for decay to the time of injection, by applying a system calibration factor (counts per second (cps)/pixel/µCi ^99m^TcO_4_/ml) determined by imaging a mouse-size (25 ml) cylindrical phantom filled with an aqueous solution of ^99m^TcO_4_ with a precisely measured activity concentration and imaged in the same manner as the mice.

### Radiopharmaceuticals

^124^I, ^131^I and ^99m^TcO_4_ were obtained from MSKCC's radiopharmacy. The maximum specific activities for the ^124^I and ^131^I compounds were ∼140 µCi/mouse and ∼0.5 µCi/well, respectively. Activity of ^99m^TcO_4_ ranged from 500–900 uCi/mouse.

### Statistical analysis

P values were generated for radiouptake assay comparisons using Dunnett's test, and for imaging radiouptake using the Tukey multiple comparisons test [49]. P<0.05 was considered significant.
